# Regulation of Prostate Androgens by Megalin and 25-hydroxyvitamin D Status: Mechanism for High Prostate Androgens in African American Men

**DOI:** 10.1158/2767-9764.CRC-22-0362

**Published:** 2023-03-03

**Authors:** Jason Garcia, Kirsten D. Krieger, Candice Loitz, Lillian M. Perez, Zachary A. Richards, Yves Helou, Steve Kregel, Sasha Celada, Clementina A. Mesaros, Maarten Bosland, Peter H. Gann, Thomas E. Willnow, Donald Vander Griend, Rick Kittles, Gail S. Prins, Trevor Penning, Larisa Nonn

**Affiliations:** 1 Department of Pathology, University of Illinois at Chicago, Chicago, Illinois.; 2 University of Illinois Cancer Center, Chicago, Illinois.; 3 Department of Systems Pharmacology & Translational Therapeutics, University of Pennsylvania Perelman School of Medicine, Philadelphia, Pennsylvania.; 4 Center of Excellence in Environmental Toxicology, Perelman School of Medicine, University of Pennsylvania, Philadelphia, Pennsylvania.; 5 Max Delbrück Center for Molecular Medicine, Berlin, Germany.; 6 Department of Population Sciences, City of Hope, Duarte, California.; 7 Departments of Urology, Physiology and Biophysics, University of Illinois at Chicago, Chicago, Illinois.

## Abstract

Vitamin D deficiency is associated with an increased risk of prostate cancer mortality and is hypothesized to contribute to prostate cancer aggressiveness and disparities in African American populations. The prostate epithelium was recently shown to express megalin, an endocytic receptor that internalizes circulating globulin-bound hormones, which suggests regulation of intracellular prostate hormone levels. This contrasts with passive diffusion of hormones that is posited by the free hormone hypothesis. Here, we demonstrate that megalin imports testosterone bound to sex hormone-binding globulin into prostate cells. Prostatic loss of *Lrp2* (megalin) in a mouse model resulted in reduced prostate testosterone and dihydrotestosterone levels. Megalin expression was regulated and suppressed by 25-hydroxyvitamin D (25D) in cell lines, patient-derived prostate epithelial cells, and prostate tissue explants. In patients, the relationships between hormones support this regulatory mechanism, as prostatic DHT levels are higher in African American men and are inversely correlated with serum 25D status. Megalin levels are reduced in localized prostate cancer by Gleason grade. Our findings suggest that the free hormone hypothesis should be revisited for testosterone and highlight the impact of vitamin D deficiency on prostate androgen levels, which is a known driver of prostate cancer. Thus, we revealed a mechanistic link between vitamin D and prostate cancer disparities observed in African Americans.

**Significance:**

These findings link vitamin D deficiency and the megalin protein to increased levels of prostate androgens, which may underpin the disparity in lethal prostate cancer in African America men.

## Introduction

Prostate cancer is the most frequently diagnosed noncutaneous cancer and the second leading cause of cancer deaths for men in the United States (1). African American (AA) men have 1.65-fold higher incidence and 2.1-fold higher mortality rates than European American (EA) men (1, 2). The reason for this disparity is multifactorial and involves both biological and socioeconomic factors; however, when controlled for, AA men still present with more aggressive disease at a younger age (3). AA men are also at increased risk of vitamin D deficiency (4) due to melanin, which decreases cutaneous synthesis of cholecalciferol (pre-vitamin D) from 7-dehydrocholesterol. Cholecalciferol is converted by the liver to the main circulating form of vitamin D, 25-hydroxyvitamin D (25D), which is the prohormone for 1,25-dihydroxyvitamin D (1,25D), a steroid hormone essential for normal human physiology and calcium homeostasis (5). Thus, vitamin D status is dependent on vitamin D supplementation, sun exposure, and skin pigmentation (4).

Associations between circulating levels of 25D and prostate cancer incidence have shown mixed results yet are more consistently found to be inversely correlated with risk of aggressive prostate cancer (6–11). Numerous anticancer activities have been demonstrated for vitamin D metabolites *in vitro* and *in vivo* (10, 12, 13). Vitamin D supplementation studies have shown promising effects, despite challenges. The VITAL (VITamin D and OmegA-3 TriaL), of over 25,000 male and female patients, did not show a reduction in prostate cancer incidence, but the study only had 192 cases of prostate cancer overall with 20 cases of metastatic prostate cancer (14). However, when examined by race, AA men had lower hazard ratios for prostate cancer in vitamin D intervention arm (14), although not significant due to very low number of events. Active surveillance patients with high 25D had a decrease in prostate specific antigen (PSA) after 9 months of supplementation (15). Furthermore, several studies demonstrate circulating levels of 25D are inversely correlated with prostate cancer aggressiveness (6–11) and the ratio of 1,25D to 25D associates with reduced risk of prostate cancer aggressiveness in AA (16). Thus, differences in vitamin D status are hypothesized to contribute to the disparity in prostate cancer incidence in AA men.

25D and other hormones are thought to follow the free hormone hypothesis which posits that intracellular concentrations of hormones are dependent on passive diffusion of “free” hormones not bound by serum globulins (17). However, we previously reported that circulating and prostate tissue levels of vitamin D metabolites did not correlate, indicating that passive diffusion of unbound hormones does not drive prostate concentrations (18). Consistent with a hormone import mechanism, we further demonstrated that prostate epithelial cells express megalin, an endocytic receptor encoded by *LRP2,* with a well-characterized function of binding and internalizing globulin-bound 25D and other hormones from the glomerular filtrate (19, 20). We further showed that prostatic expression of the megalin gene (*LRP2*) was negatively correlated with 25D levels only in AA men (18). *LRP2* was also positively correlated with the percentage of West African ancestry in the cohort (18). These findings suggest that the free hormone hypothesis may not apply to the prostate and that a compensatory mechanism may increase prostate megalin levels when systemic levels of 25D are deficient.

Megalin also binds to and internalizes sex hormone-binding globulin (SHBG), the serum transporter of testosterone (T). Import of SHBG-bound T by megalin occurs most notably in kidney cells, but import of SHBG has also been shown in LNCaP prostate cancer cells (21). Circulating T concentrations were not correlated with intraprostatic concentrations, further supporting an alternative to passive diffusion (22). Megalin knockout in mice is perinatal lethal and the pups exhibit defects in the maturation of their reproductive organs, suggesting dysregulation of sex hormones (23). In one study, polymorphisms in the megalin gene *LRP2* are associated with prostate cancer recurrence, prostate cancer–specific mortality, and the effectiveness of androgen deprivation therapy (24).

Here, we examined megalin as a mechanism to import T into the prostate and the regulation of megalin by vitamin D metabolites. This mechanism is highly relevant to prostate cancer disparities, given that androgens contribute to prostate cancer pathogenesis and AA men are more likely to be 25D deficient. Here, we show mechanistic examination of T transport by megalin in prostate cells, an inducible prostate-specific knockout mouse model, and patient prostate tissue explants. We further validated the findings in clinical prostate specimens.

## Materials and Methods

### Patient Sera and Prostate Tissue

Patient specimens were deidentified from the three biorepository cohorts. Each biorepository acquired written informed consent from the patients in accordance with recognized ethical guidelines (e.g., Declaration of Helsinki, CIOMS, Belmont Report, U.S. Common Rule) in Institutional Review Board (IRB)-approved protocols. The specimens were deidentified for this study, thus use of the specimens was determined to not fit the definition of human subjects research by the University of Illinois Chicago (UIC) IRB (2013-0341).

Cohort 1 included paired fresh-frozen prostate tissue and serum 56 patients; 30 (*N* = 21 AA, *N* = 9 EA) from the UIC Hospital (Chicago, IL), and 26 (*N* = 8 AA, *N* = 18 EA) from the Cooperative Human Tissue Network (CHTN) Western Division at Vanderbilt University (Nashville, TN). The CHTN specimens were acquired under exemption (IRB 2018-0281). UIC fresh-frozen prostate specimens were collected from radical prostatectomy patients under IRB 2004-0679 and 2015-1294. Cohort 2 included only fresh-frozen prostate tissue from UIC patients collected under IRB 2004-0679 (*N* = 117 AA, *N* = 29 EA). Cohort 3 comprised serum only (*N* = 46 AA, *N* = 51 EA) and was acquired from the Prostate Cancer Biorepository Network (PCBN) and from UIC (IRB 2017-0807, do not have matched frozen tissues). For all cohorts, race was self-declared, the patients had localized prostate cancer, node-negative disease without prior chemotherapy or hormone therapy. Frozen tissues in cohorts 1 and 2 were from benign areas of the peripheral zone, as confirmed by an adjacent frozen section.

### Cell Lines

HEK293, LNCaP, and 22Rv1 cells were purchased from ATCC, 957E-hTERT cells were generously donated by John Isaacs and MDA-PCa-2b cells were gifted by Donald Vander Griend. LNCaP, 22Rv1, and 957E-hTERT cell lines were from White patients and MDA-PCa-2B were derived from an AA patient. Cell lines were authenticated by short tandem repeat analyses prior to use and tested for *Mycoplasma* every 6 months. PrE-AA1 and PrE-AA2 are primary patient-derived epithelial cells established from two AA patients with prostate cancer using previously reported methods (13, 25) and are cultured in prostate cell growth medium (catalog no. CC-3165, Lonza). PrE cell lines are used on secondary passage and passaged one time. Immortalized cell lines (HEK293, LNCaP, 22Rv, MDA-PCa-2B, 957E-hTERT) were cultured no longer than 2 months. 957E-hTERT cells were maintained in keratinocyte serum-free medium (catalog no. 17-005-042, Thermo Fisher Scientific). HEK293 cells were maintained in DMEM (catalog no. 21063-029 Gibco) supplemented with 10% [volume for volume (v/v)] FBS (catalog no. 26140-079, Gibco). LNCaP and 22Rv1 cells were maintained in phenol-free RPMI medium (catalog no. 11835055, Thermo Fisher Scientific) supplemented with 10% (v/v) FBS. MDA-PCa-2B cells are grown in BRFF-HPC1 (catalog no. P9054 US Biological) with 20% FBS. Cells were switched to 5% (v/v) charcoal-stripped FBS (catalog no. F6765-500ML, Millipore-Sigma) overnight and serum starved for 1 hour prior to experimentation. All cells were cultured at 37°C and 5% CO_2_. All the cells are described in Supplementary Table S1.

### RNA Isolation and qRT-PCR

RNA was isolated using TRIzol reagent (catalog no. 15596-018, Thermo Fisher Scientific). RNA concentration and quality were determined by measuring the absorbance ratio at 260/280 nm using a NanoDrop One spectrophotometer (Thermo Fisher Scientific). Total RNA (500 ng) was reverse transcribed using the High-Capacity cDNA Reverse Transcription Kit (catalog no. 4368814, Applied Biosystems). The resulting cDNA was used for quantitative PCR amplification on a QuantStudio6 machine (Thermo Fisher Scientific) using gene-specific primers (Supplementary Table S2) and FastStart Universal SYBR Green master mix (catalog no. 04913850001 Millipore-Sigma). Reactions were run in duplicate and relative *C*_t_ values were normalized and calculated independently using the −∆∆*C*_t_ method for the expression of the housekeeping genes *HPRT1* and *RPL13A* (all primers are listed in Supplementary Table S2)*.*

### Western Blotting

Cells were grown to 80% confluence, and protein lysates were collected in cell lysis buffer (catalog no. 9803, Cell Signaling Technology). Protein (30 μg) was electrophoresed on a Bis-Tris protein gel (catalog no. NP0335BOX NuPAGE) and transferred to a polyvinylidene difluoride membrane. Membranes were blocked for 1 hour using Odyssey Blocking Buffer (catalog no. 927-60003 LI-COR) and probed with anti-megalin rabbit mAb (1:1,000; catalog no. M02463, Boster Bio) and anti-actin (1:1,000; catalog no. 4499S, Cell Signaling Technology), and with secondary antibodies against rabbit and mouse (catalog no. 926-68071, LI-COR). Blots were imaged using an Odyssey CLx imaging system (LI-COR).

### T and SHBG Treatments

LNCAP and 22Rv1 cells at 80% confluency were incubated with 25 nmol/L T ± 125 nmol/L human SHBG (catalog no. SHBG-8259H, Creative BioMart) and 1 μmol/L receptor-associated protein (MEG-Inh; catalog no. BML-SE552-0100, Enzo Life Sciences) for 16 hours. T and SHBG were preincubated for 30 minutes before addition to cells. Cells were pretreated with megalin inhibitor for 1 hour before hormone addition.

### SHBG-555 Internalization

Recombinant human SHBG (catalog no. SHBG-8259H, Creative BioMart) was directly labeled with Alexa Fluor-555 using a protein conjugation kit (catalog no. A20174, Thermo Fisher Scientific) according to the manufacturer's protocol. Aliquots of the globulin conjugate SHBG-555 were stored at −20°C until use. Cells were grown to 70% confluence in 8-well chamber slides and incubated with SHBG-555 alone, SHBG-555 +T, or MEG-Inh + SHBG-555 + T, as described above. After 4 hours, the cells were counterstained with Alexa Fluor 647 phalloidin (F-actin; catalog no. A22287, Thermo Fisher Scientific) and DAPI (Thermo Fisher Scientific) and visualized by confocal microscopy.

### *Lrp2*-flox/Pb-MerCreMer Mice

All procedures involving animals in this study were approved by the University of Illinois at the Chicago Office of Animal Care and Institutional Biosafety. Transgenic mice harboring the probasin promoter driving MerCreMer were acquired from Jackson Laboratory [ProbasinBAC-MerCreMer or C57BL/6-Tg(Pbsn-cre/Esr1*)14Abch/J, strain 020287]. Generation of mice with *loxP* sites flanking *Lrp2* exons 71 through 75 (*Lrp2*^fl/fl^) has been described previously (26). *Lrp2*^fl/fl^ mice were crossbred with homozygous Pb-MerCreMer (*Cre*^+/+^) mice. F1 cross progenies were mated to generate *Lrp2*^fl/fl^/*Cre*^+/+^ mice. Mice were genotyped using Transnetyx and injected with tamoxifen (TAM; 50 mg/kg) at two stages of development (P10 or 5 weeks). Control TAM-injected mice were *Lrp2*^fl/fl^ or *Cre*^+/+^. To confirm recombination, DNA was isolated from tail snips and prostate cell pellets using a DNeasy Blood & Tissue kit (catalog no. 69504 Qiagen), followed by PCR with DreamTaq Green PCR master mix (catalog no. EP0701, Thermo Fisher Scientific) using primers spanning exons 71–75 and primers spanning exons 76–77 as a control. PCR products were imaged using agarose gels. The primers are listed in Supplementary Table S2.

### DHT and T Quantitation for Mice and Patient Cohorts 2 and 3

A protocol similar to that described by Higashi and colleagues was followed (27) at the University of Pennsylvania Perelman School of Medicine (Philadelphia, PA). Briefly, the internal standard mix (500 pg each of T-IS, DHT-IS, E1-IS, and E2-IS and 100 pg of 3α- and 3β-diol) were added to 0.6 mL of 0.1 mmol/L PBS in a homogenization vial kept in ice. Frozen tissue (∼20 mg) was cut on a tile in dry ice with a blade kept in dry ice and added directly to the homogenization vial. The tissue was homogenized twice for 10 minutes on ice in a bullet blender. The homogenate was transferred to a borosilicate tube and ethyl ether (4 mL) was added and shaken for 30 minutes, followed by incubation for 2 hours at 50°C with shaking at 4°C overnight. The specimen was then centrifuged at 1,500 × *g* for 10 minutes. The upper organic phase was transferred to a new borosilicate tube using a glass pipette. The organic phase was then dried under nitrogen. Specimens were stored at −20°C before derivatization and LC/MS analysis, as described previously. With each batch of specimens, we prepared a new calibration curve extracted from charcoal-stripped serum and calculated the precision for each individual point to be within 15% of the theoretical amount spiked. The slope coefficients varied with less than 15% between batches. We also prepared at least five individual pooled specimens that were processed separately, to be used as quality controls (QC). The QC samples had a coefficient of variability under 15% for each batch.

### Luciferase Reporter Assays

Cells at 70% confluence were transfected with luciferase plasmids, and luciferase activity was measured after 48 hours using the dual-luciferase reporter assay system and GloMax 20/20 (Promega). pRL-null *Renilla* plasmid was cotransfected at 0.4 pg/μL to control for transfection efficiency. For *LRP2* promoter, 0.2 ng/μL *LRP2* promoter-driven *Renilla* luciferase reporter (S712992, Switchgear Genomics) and 0.4 pg/μL PGL4.50 *Photinus pyralis* (E310, Promega) luciferase reporter were simultaneously treated with 10 nmol/L 25D or 10 nmol/L T. *Renilla* luciferase relative light units are defined as the ratio of *Renilla* to *Photinus* activity.

### *Ex Vivo* Prostate Tissue Slice Culture

Fresh prostate tissue was obtained from radical prostatectomy patients at UIC with informed consent (IRB 2007-0694). Tissue from a 5-mm punch was sliced into 300-μm sections using an Alabama Tissue Slicer (Alabama Research and Development), placed on titanium alloy inserts within a 6-well plate, and maintained in 2.5 mL of KSFM supplemented with 5% (v/v) charcoal-stripped FBS and 50 nmol/L R1881 (PerkinElmer; ref. 28). Slices were cultured overnight, rotating at a 30° angle, at 37°C with 5% CO_2_. Alternating slices were collected for RNA extraction and formalin fixation. For gene expression analysis, RNA isolation and qRT-PCR were performed as described above. Only slices with confirmed benign epithelial content (high expression of KRT8 and undetectable PCA3 by qRT-PCR) were included in the analyses.

### IHC of Tissue Slice Explants

Formalin-fixed paraffin-embedded slices were sectioned into 5 μm sections, deparaffinized, processed for steam antigen retrieval (10 mmol/L sodium citrate, 0.05% Tween 20, pH 6), and stained with anti-megalin (1:500; ab76969, Abcam) overnight at 4°C. A rabbit secondary antibody HRP/DAB kit was used for visualization with hematoxylin counterstaining (ab64261, Abcam).

### Hormone Measurement in Specimens from Patient Cohort 1

Tissue and serum in cohort 1 was analyzed by the UIC Research Resource Center Mass Spectrometry core.

#### Calibration Curve

Standard compounds for T and DHT and the internal standard (IS) d3T were purchased from Cerilliant. Nine calibrators (0.0625, 0.125, 0.25, 0.5, 1, 5, 10, 50, and 100 ng/mL in methanol) were used to establish calibration curves with the spiked-in IS. The curves were fitted by linear regression with a weighting factor of 1/*x*.

#### Specimen Preparation and Extraction

Fresh-frozen patient prostate tissue specimens (100 mg) and ISs were mixed and bead homogenized using a Mikro-Dismembrator II (Handelskontor Freitag) prior to extraction. Extraction was performed three times with hexane:ethyl acetate [60:40 (vol:vol)]. The organic layer from each extraction was collected, combined, and dried under nitrogen. The residue was reconstituted in methanol:water [20:80 (vol:vol)] and subjected to solid-phase extraction using an ISOLUTE C18 SPE cartridge (100 mg, 1 mL), following the manufacturer's protocol. The final eluate was dried before LC/MS analysis. Human serum specimens were extracted using the same procedure.

#### LC/MS-MS Analysis

Quantification of T and DHT was performed using an SCIEX Qtrap 6500 spectrometer coupled with an Agilent 1290 ultra-performance liquid chromatography (UPLC) system. The dried specimen was reconstituted in methanol and resolved using a Waters ACQUITY UPLC BEH C18 column (1.7 μm, 2.1 × 100 mm) maintained at 45°C at a flow rate of 450 μL/minute. The elution started with 60% mobile phase A (5% methanol in water, 0.1% formic acid), followed by a linear gradient increase in mobile phase B (acetonitrile with 0.1% formic acid) from 40% to 80%. MS data were acquired by multiple reaction monitoring in positive mode with an electrospray ionization source voltage of 5.0 kV and a temperature of 450°C. T, DHT, and D3T were detected by monitoring their transitions to the signature product ions 289>97 (T), 291>255 (DHT), and 292>97 (D3T), respectively. With each batch of specimens, we prepared a new calibration curve extracted from charcoal-stripped serum and calculated the precision for each individual point to be within 15% of the theoretical amount spiked. The slope coefficients varied with less than 15% between batches. We also prepared at least five individual pooled samples that were processed separately, to be used as QC. The QC samples had a coefficient of variability under 15% for each batch.

Data were analyzed using the Analyst software.

#### Measurement of Vitamin D Metabolites

The extraction and measurement of 25D was previously reported by our group and was done on 50 μL of serum at Heartland Assays (18).

### Tissue Microarray Immunofluorescent Staining and Analysis

The tissue microarray (TMA) contained 118 prostate biopsy cores from 29 patients (20 AA, 9 EA) with two benign and two cancer cores from each patient. A board-certified pathologist reviewed each core to confirm the cancer grade mark regions for exclusion if they contained artifacts or benign areas intermixed with cancer. Sections (5 μm) were incubated with rabbit polyclonal anti-megalin (catalog no. ab76969, Abcam) diluted 1:100 and mouse monoclonal anti-panCK (catalog no. AE1/AE3) diluted 1:2,000, followed by incubation with secondary antibodies Alexa Fluor 488 goat anti-rabbit diluted 1:200 and Alexa Fluor 555 goat anti-mouse diluted 1:200 (catalog no. A21428 and A-21422, Life Technologies) and counterstaining with DAPI. Sections were scanned at 20X on a Vectra3 multispectral imaging system (Akoya Biosciences). Epithelial areas were identified and segmented by machine learning using the panCK marker and HALO software (Indica Labs) and adjusted manually to ensure accuracy. Epithelial megalin fluorescence intensity was quantified and reported as the average intensity per pixel of the segmented area of each core using the Inform software. The Mann–Whitney *U* test was used to compare benign cores to tumor cores for all men, EA only, and AA only.

### *LRP2* Expression in Public Datasets

RNA-sequencing datasets for prostate cancer tumors were identified and data exported from cBioPortal (29). Datasets were selected if they contained mRNA expression, greater than 200 patients, postoperative Gleason and/or benign/normal prostate for comparison. Datasets with biochemical recurrence (BCR) were also included and are shown in the Supplementary figures. *LRP2* expression was analyzed using ANOVA with Kruskal–Wallis or Fisher for multiple comparisons.

### Statistical Analysis

The statistical analysis methods used in each experiment are detailed in the figure legends and Materials and Methods section.

### Data Availability

All data generated or analyzed during this study are included in this published article (and its Supplementary Data). Transgenic mice are available as frozen embryos upon request at the cost of the requestor.

## Results

### Prostate Cells Express Megalin and Import SHBG-bound T

**FIGURE 1:**
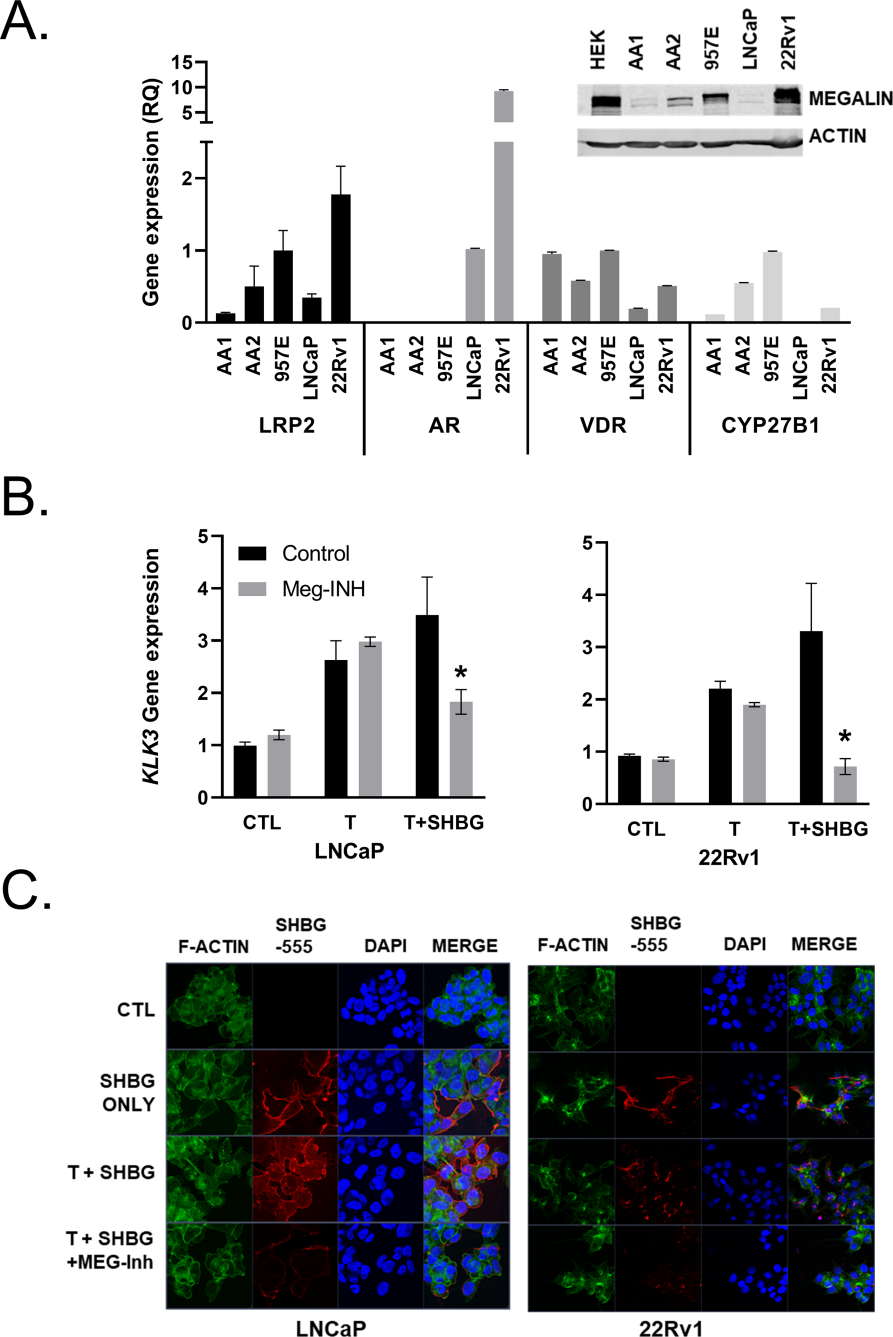
Prostate cells express megalin and import SHBG-bound T. **A,** Gene expression of *LRP2, AR, VDR,* and *CYP27B1* in a panel of prostate cell lines as shown by qRT-PCR shown as relative quantitation to *HPRT1*. Error bars are SEM. (**A**, inset) Western blot analysis for megalin in prostate cell line panel. **B,** Regulation of *KLK3* (PSA gene) expression after 24 hours following treatment with vehicle control (CTL), 50 nmol/L T alone (T), 50 nmol/L T preincubated with 500 nmol/L SHBG (SHBG), or T + SHBG in cells preincubated with 1 μmol/L MEG-Inh in LNCaP and 22Rv1 prostate cancer lines. **C,** Visualization (× 63) of SHBG-555 (red) import into cells with DAPI (blue) nuclear and F-actin (green) cytoskeletal counterstains. Statistical analysis was performed using a one-way ANOVA with a two-stage linear step-up procedure of Benjamini, Krieger, and Yekutieli for multiple comparisons; *, *P* < 0.05 for comparison with CTL, all graphs represent mean ± SEM of three or more individual experiments with two replicates per experiment.

Because we previously detected megalin in prostate tissues, megalin transcript (*LRP2*) and protein expression were quantified in prostate cell lines. Both mRNA and protein were detected in benign primary patient-derived prostate epithelial cells (PrE-AA1 and PrE-AA2), immortalized benign prostate epithelial cells (957E-hTERT), and prostate cancer cell lines (LNCaP and 22Rv1; Fig. 1A). MDA-PCa-2B cells, the only line derived from an AA patient, and AR-negative PC3 cells did not express detectable *LRP2* (Supplementary Fig. S1B). All cell types expressed the vitamin D receptor (VDR); however, benign PrE cells (AA1, AA2, and 957E-hTERT) lacked androgen receptor (AR) expression (Fig. 1A). Prostate cancer cells (LNCaP and 22Rv1) showed low to no expression of CYP27B1 (encoding a vitamin D 25-hydroxylase; Fig. 1A) and were unable to metabolize 25D to the active hormone 1,25D, as shown by the lack of CYP24A1 (encoding vitamin D 24-hydroxylase) induction by 25D (Supplementary Fig. S1A).

LNCaP and 22Rv1 prostate cancer cell lines were used to examine testosterone (T) import and AR activity *in vitro*, as they express AR and have differential expression of megalin. When treated with T alone and SHBG-T, both LNCaP and 22Rv1 cells showed AR activation, as evidenced by the increased *KLK3* mRNA levels (Fig. 1B). SHBG was added in 20-fold excess and incubated with T for 30 minutes before treating cells to ensure thorough hormone-globulin binding. To inhibit megalin, the cells were preincubated with receptor-associated protein (MEG-INH; ref. 30; Fig. 1B). Both LNCAP and 22Rv1 cells exhibited decreased *KLK3* gene expression when cells were pretreated with MEG-Inh before hormone treatment; however, the magnitude of inhibition was higher in 22Rv1 cells, which express more megalin than LNCaP at basal levels. Preincubation with MEG-Inh did not block the response to added T alone, demonstrating its specificity to SHBG-T. To visualize SHBG/T internalization, we used Alexa Fluor 555-labeled human SHBG (SHBG-555). SHBG-555 was localized to the plasma membrane of LNCaP and 22Rv1 cells in response to SHBG and SHBG + T treatment (Fig. 1C). The addition of SHBG-T resulted in greater internalization and punctate patterns than SHBG treatment alone, and MEG-Inh blocked the internalization of SHBG. These data demonstrate that SHBG-bound T enters prostate cells in a megalin-dependent manner.

### Knockout of *lrp2* in Mouse Prostate Reduces T

**FIGURE 2:**
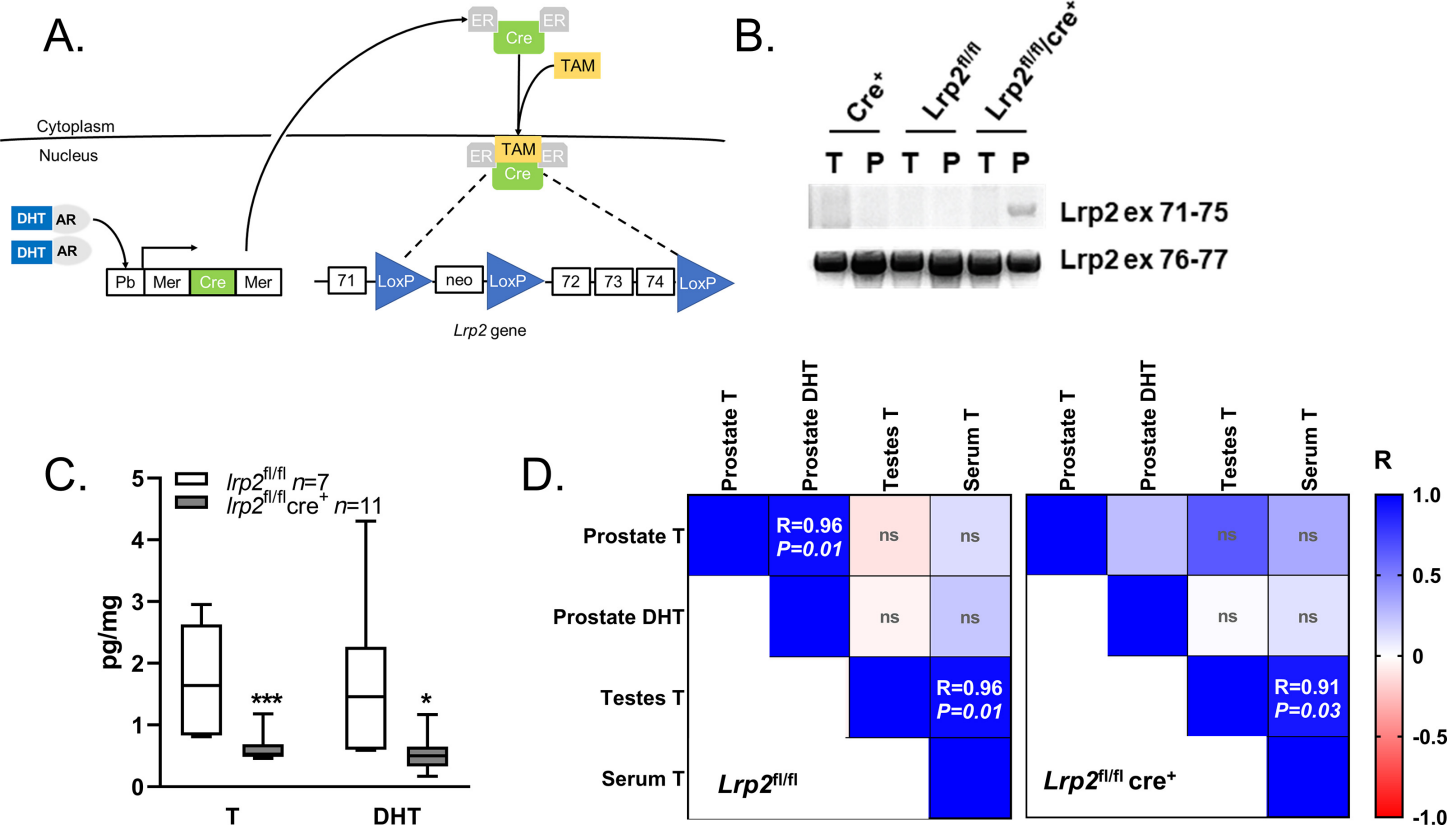
Loss of *Lrp2* in the mouse prostate reduces prostatic androgen levels. **A,** Mouse model of conditional knockout of *Lrp2* in prostate epithelium. **B,** Recombination of *Lrp2* exons 71–75 after TAM treatment in prostate DNA (P), but not in tail DNA (T), only in bitransgenic mice. PCR for exons 76–77 was used as the positive control. **C,** Prostate levels of T and DHT quantified by LC/MS-MS for *Lrp2*^fl/fl^ and *Lrp2*^fl/fl^/*Cre*^+/+^. The graphs show the mean with maximum–minimum bars. **D,** Heatmap of Pearson correlation coefficients (*R*) for tissue and serum androgens in P10-TAM mice (*Lrp2*^fl/fl^*n* = 5, *Lrp2*^fl/fl^/*Cre*^+/+^*n* = 6). The *P* value is shown within each cell; ns, not significant.

To determine whether prostate T levels are affected by the absence of megalin, we created an inducible prostate-specific knockout of *Lrp2,* because obligate *Lrp2* knockout causes perinatal lethality in most affected animals (26, 31). A TAM-inducible prostate-specific knockout of *Lrp2* was generated by crossing an *Lrp2*-floxed mouse (32) with a probasin-driven TAM-inducible Cre recombinase mouse (Pb-MerCreMer; ref. 33; Fig. 2A). No breeding problems were encountered with the homozygous bitransgenic line. *Lrp2*^fl/fl^/*Cre*^+/+^ mice and control (*Lrp2*^fl/fl^ only and *Cre*^+/+^ only) mice were injected with TAM at 5 weeks of age, which resulted in recombination of genomic DNA only in the prostate and not in mouse tails (Fig. 2B). Whole prostates, testes, and sera were collected at 24 and 32 weeks of age for androgen measurement by LC/MS-MS. Prostate T and DHT levels were significantly lower in *Lrp2*^fl/fl^/*Cre*^+/+^ mice than in the control mice (Fig. 2C). Serum T and testes T levels were tightly correlated in all mice, supporting testes as the source of serum T. However, neither prostate T nor DHT was significantly correlated with serum T in control and *Lrp2*^fl/fl^/*Cre*^+/+^ mice (Fig. 2D), suggesting that prostate T levels are not due to passive diffusion from the serum. In addition, prostate T and DHT were only significantly correlated in the control *Lrp2*^fl/fl^ group, suggesting that the regulation of T to DHT is different in prostates lacking *Lrp2*. Serum DHT was undetectable in most mice, consistent with T as the primary circulating hormone. The relationship between serum and tissue hormone levels supports a regulated transport mechanism of T into the prostate, rather than passive diffusion of serum T. Although the differences in prostate androgens were significant, there were no differences in prostate histology, prostate weight, or fertility (Supplementary Fig. S2).

### Vitamin D Negatively Regulates *LRP2* Expression

**FIGURE 3:**
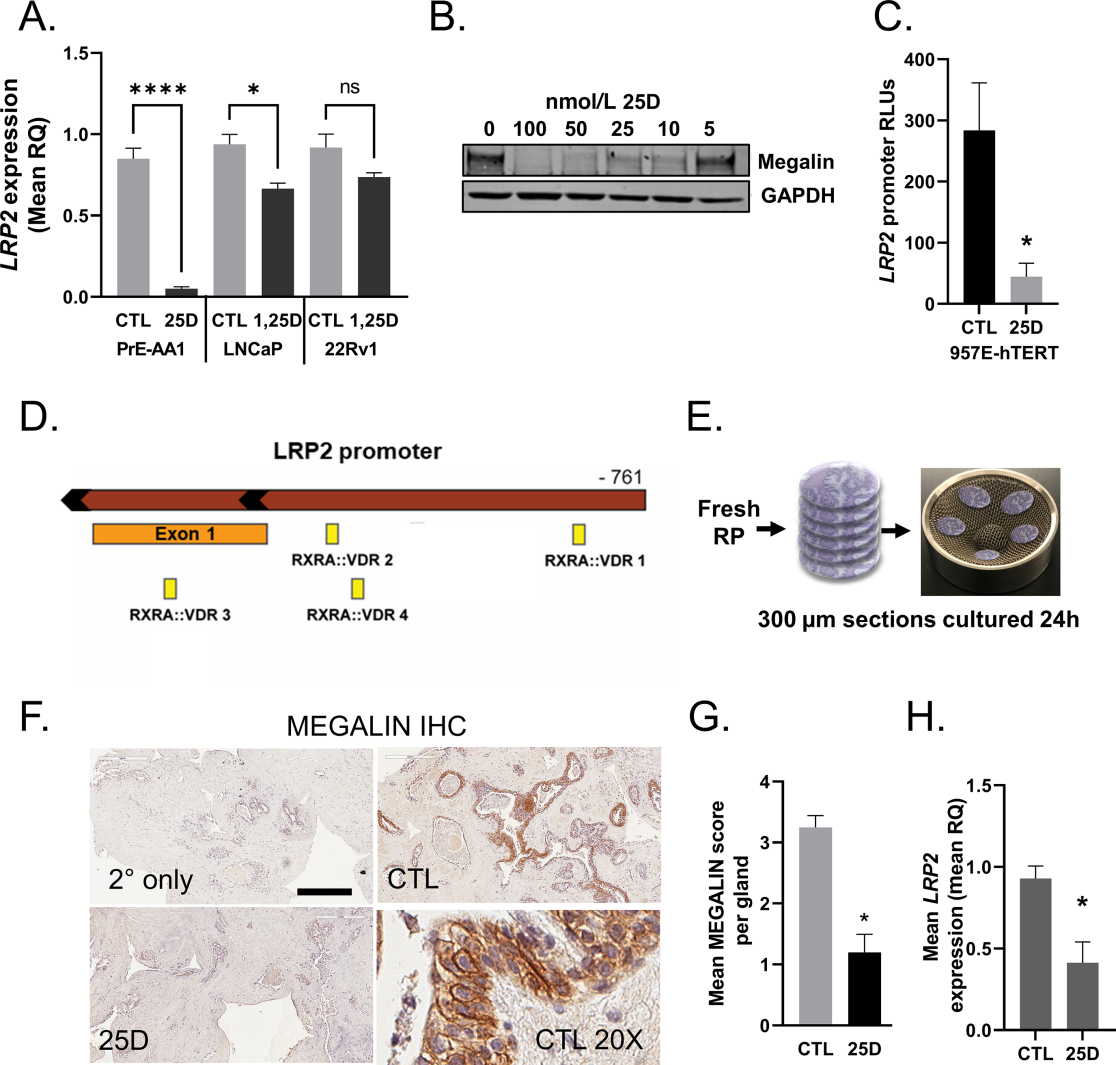
Negative regulation of *LRP2* and megalin proteins by vitamin D in prostate cells and tissue slice explants. **A,***LRP2* expression following 24 hours of treatment with 50 nmol/L 25D in PrE-AA1, 50 nmol/L 1,25D in LNCaP and 50 nmol/L 1,25D in 22Rv1 cells. **B,** Immunoblot for megalin after 48 hours of 25D treatment of PrE-AA1 cells. **C,** Activity of a custom *LRP2* promoter luciferase construct after 24 hours of 50 nmol/L 25D treatment in 975E-hTERT cells; RLU, relative luciferase units normalized to the transfection control. **D,***LRP2* promoter contains the RXR:VDR binding motifs. **E,***Ex vivo* prostate tissue slice workflow. **F,** Images and quantification of megalin protein by IHC (**G**) and LRP2 gene expression in tissue slices (**H**) after 24 hours of treatment with 50 nmol/L 25D. Scale bar, 150 μm. The graph shows the mean pathologist score per gland. Graphs represent the mean ± SEM from triplicate experiments. For tissue slices, graphs show the representative experimental mean ± SD with two replicates per experiment. *P* values were determined using an unpaired *t* test.

We previously observed a negative correlation between serum 25D levels and *LRP2* prostatic expression in AA men, suggesting that *LRP2* is regulated by 25D. Therefore, we sought to characterize the effect of 25D on *LRP2* gene and megalin protein expression *in vitro*. Primary prostate epithelial cells (PrE-AA1) treated with 25D exhibited decreased *LRP2* gene and megalin protein expression (Fig. 3A and B). 1,25D-treated LNCaP and 22Rv1 prostate cancer cells also exhibited decreased LRP2 expression, but to a lesser extent than the benign cells (Fig. 3A). To assess the regulation of *LRP2* expression at the transcriptional level, we characterized the *LRP2* promoter activity in vitro. The *LRP2* promoter was cloned into the *Renilla* luciferase reporter plasmid (*LRP2-Rluc*), which was suppressed by 25D in 957E-hTERT cells (Fig. 3C). 957E-hTERT cells were used for luciferase experiments because they can be transfected at high efficiency, whereas PrE cells are difficult to transfect. 957E-hTERT cells are immortalized prostate cells that do not express AR and are phenotypically similar to PrE cells (34). VDR forms an obligate heterodimer with the retinoid X receptor (RXR) and binds to the vitamin D response elements. Predictive analysis of the *LRP2* promoter fragment identified multiple RXR:VDR motifs, indicating VDR may bind to these transcriptional regulatory sites (Fig. 3D; Supplementary Fig. S3). These transcriptional analyses support that *LRP2* expression is regulated by 25D.

Megalin expression was examined *ex vivo* in benign human prostate tissue slice cultures (Fig. 3E), which express all components of androgen and vitamin D activation/response pathways, including *CYP27B1* and *VDR*, *LRP2*, *SRD5A* (encoding a T to DHT conversion enzyme), and *AR* (Supplementary Fig. S4A). Hormone responsiveness of the tissue slices was demonstrated by the robust induction of *CYP24A1* and *KLK3* gene expression by 25D and T, respectively, alone or in the presence of their serum-binding globulins, DBP, and SHBG (Supplementary Fig. S4B and S4C). Megalin protein and *LRP2* expression were decreased in tissue slices treated with 25D (Fig. 3F–H), consistent with our observations in cell lines and the relationships we previously observed between serum 25D and prostate *LRP2* in patient data (18). Expression of megalin protein was markedly low in areas of cancer in the explants and not further decreased with 25D treatment (Supplementary Fig. S4D). These findings demonstrate vitamin D-mediated negative feedback on *LRP2* expression.

### Intraprostatic DHT is Higher in AA Men and Inversely Correlates with Vitamin D Status

**FIGURE 4:**
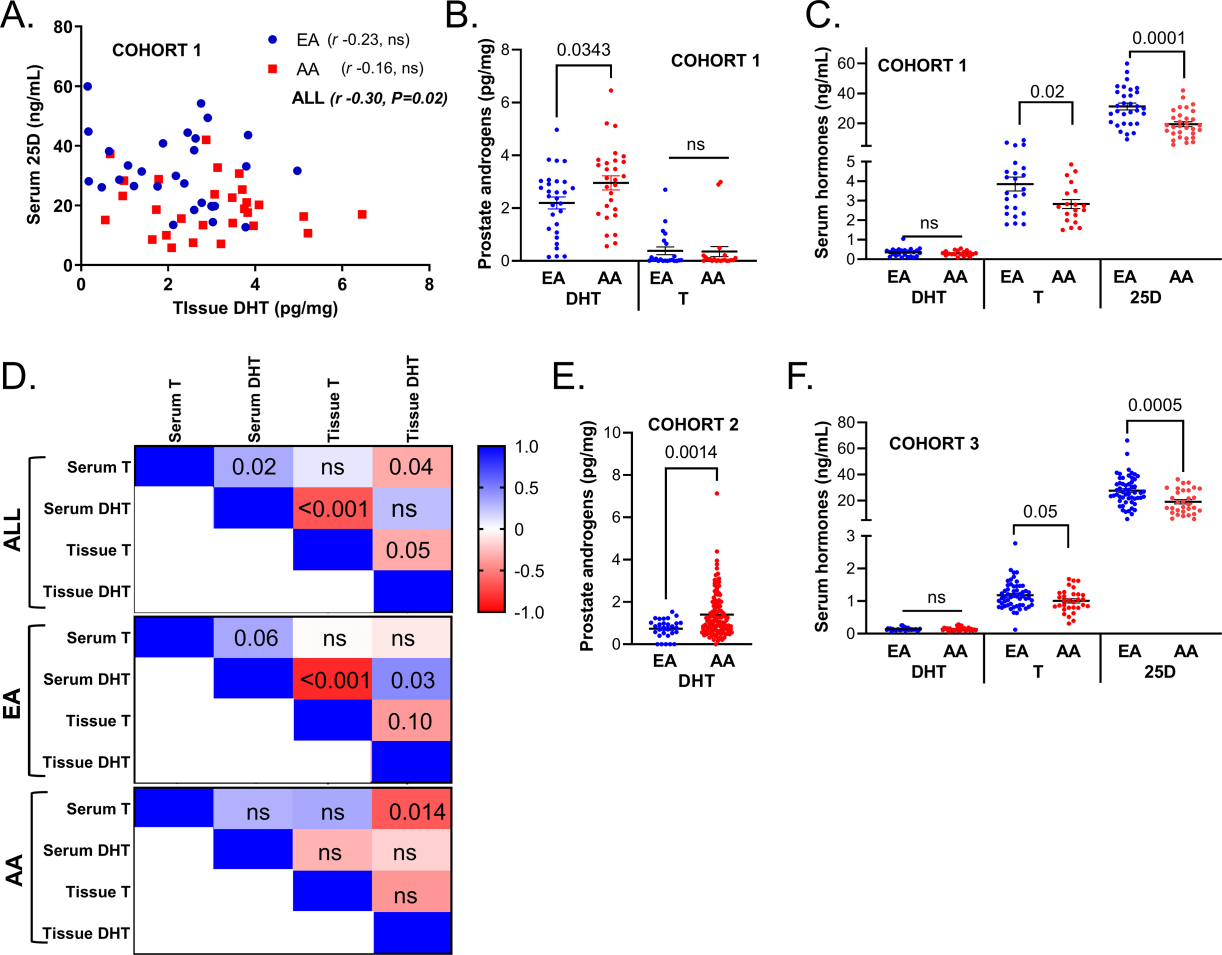
Ancestry-specific differences in androgen levels and relationships between DHT and 25D. **A,** Correlation between serum 25D and prostate DHT in EA (*n* = 29) and AA (*n* = 28) men in cohort 1. Correlation values (*r*) were determined using Spearman rank test. **B,** Prostate tissue levels of T and DHT in cohort 1. **C,** Serum levels of T, DHT, and 25D in cohort 1. **D,** Spearman correlations (R shown in heatmap) between serum and prostate tissue concentrations of hormones. *P* values shown within cells. **E,** Prostate tissue levels of DHT in cohort 2 (EA *n* = 13, AA *n* = 82). **F,** Serum levels of DHT, T, and 25D in cohort 3 (EA *n* = 33, AA *n* = 28). All hormone measurements were quantified by LC/MS-MS. Graphs represent mean with 95% confidence interval. The *P* values were determined by unpaired two-tailed *t* test.

Negative regulation of megalin by vitamin D suggests that vitamin D deficiency may lead to megalin upregulation and, subsequently, increased prostate import of SHBG-bound T. To test this hypothesis, we examined the relationship between these hormones in three cohorts of patients. DHT was quantified in frozen radical prostatectomy tissues from a cohort of patients with prostate cancer for whom we had previously measured vitamin D metabolites (18). Vitamin D status, as measured by serum 25D level, negatively correlated with intraprostatic DHT (Fig. 4A) in all patients but was not significant when analyzed separately by self-declared race. In this cohort, AA patients had higher prostate levels of the active hormone DHT than EA men (Fig. 4B). DHT was the predominant androgen in the prostate, whereas T levels were low or undetectable in the majority of patients (Fig. 4B), supporting the metabolism from T to DHT once in the tissue. In the serum, T was the dominant androgen and was slightly lower in AA men than in their EA counterparts, which was similar to their 25D status (Fig. 4C). Analyses of androgens by Gleason sum and age did not show significant differences (Supplementary Fig. S5A–S5D).

The free hormone hypothesis would be supported by the positive correlations between serum and tissue androgen levels, but this is not what we observed. Correlation analyses between prostate tissue and serum androgen levels showed that only EA men had a positive correlation between serum DHT and tissue T (Fig. 4D). In contrast, an inverse correlation was observed between serum T and prostate tissue DHT in the overall cohort and in AA men (Fig. 4D). There was a very high correlation between serum DHT and tissue T in EA men only, which may be due to the low levels of both in the patients (Fig. 4D).

We followed up on these findings in two additional tissue and serum cohorts; however, these were not matched. Prostate DHT was quantified in cohort 2 of prostate cancer patients and we observed a similar difference in tissue DHT between the AA and EA groups (Fig. 4E). However, there were very few EA men in cohort 2, and we did not have matched serum. Cohort 3 was serum only, without matched tissue, and the levels of hormones were similar to those in cohort 1, with lower T and 25D in AA men than those of White men (Fig. 4F).

Overall, the inverse correlation between serum 25D and intraprostatic DHT supports the hypothesis that serum vitamin D deficiency may promote higher prostatic DHT levels. Second, the relationship between hormones in the serum and tissues differs by ancestry and does not support the free hormone hypothesis in AA men.

### Megalin and *LRP2* Levels are Lower in Prostate Cancer Tumors

**FIGURE 5:**
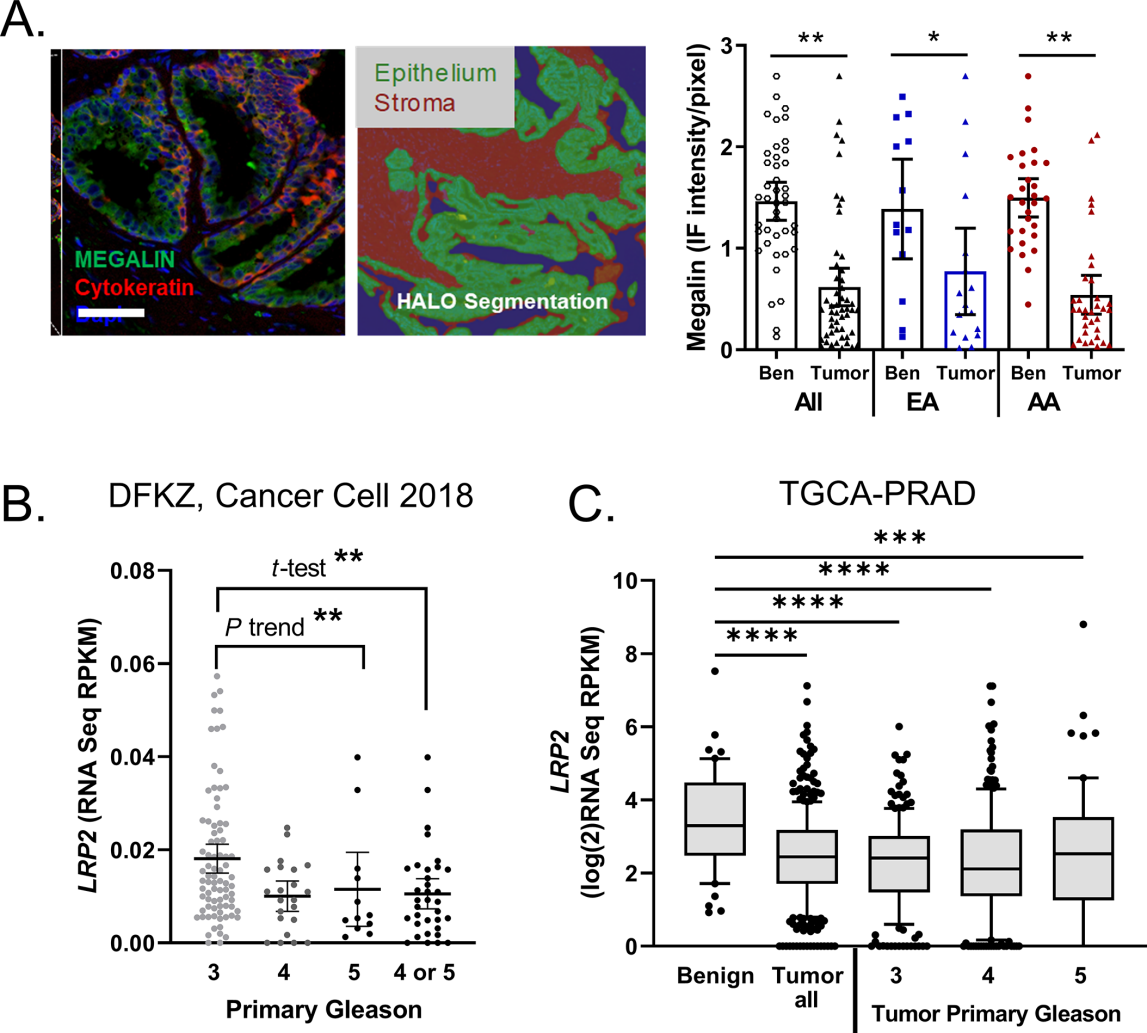
Megalin expression is dysregulated in cancer and lower with disease aggressiveness. **A,** Digital quantitation of epithelial megalin expression on a TMA consisting of 118 prostate biopsy cores from 29 patients (EA, *n* = 9; AA, *n* = 20). Epithelial regions were segmented by panCK staining, and benign and cancer regions were determined by a board-certified pathologist. Scale bar, 100 μm. Immunofluorescence (IF) intensity per pixel for megalin expressed as mean ± SEM; Ben, benign. **B,***LRP2* expression in RNAseq dataset of primary prostate cancer tumors by primary Gleason grade from Gerhauser and colleagues DHFZ dataset. Analyses by Gleason was ANOVA with Fisher multicomparisons for Gleason 3 (*n* = 82) and 4 (*n* = 24) or 5 (*n* = 12) combined. **C,***LRP2* expression in TCGA-PRAD RNAseq dataset with normal (*n* = 52) and tumor (*n* = 374) with breakdown by primary Gleason grade (Gleason 3 *n* = 206, Gleason 4 *n* = 249, Gleason 5 *n* = 63). ANOVA with Fisher multicomparisons test. *, *P* < 0.01; **, *P* < 0.001; ***, *P* < 0.001; ****, *P* < 0.0001.

To characterize LRP2 levels in clinical specimens we examined a TMA composed of prostate cores from 29 patients (20 AA, 9 EA), with four cores from each patient, two benign and two tumor regions per patient, stained for megalin. Fluorescence intensity was digitally quantified in the epithelial regions using the epithelial marker Pan-CK to segment the epithelium (Fig. 5A). Neither frozen tissues nor serum were available for patients represented in this TMA; therefore, we could not correlate the hormone levels with megalin expression. However, megalin protein levels were significantly lower in tumor areas than in benign tissues (Fig. 5A). The majority of the prostate cancer on this TMA was Gleason 3+3, with only five cases containing areas of Gleason 4, and no difference by Gleason was observed in this small cohort.

*LRP2* expression was assessed in two larger cohorts of publicly available datasets. Analyses of gene expression data from a cohort of localized prostate cancer (DFKZ; ref. 35) showed that *LRP2* levels decreased with primary Gleason grade (Fig. 5B). In The Cancer Genome Atlas Prostate Adenocarcinoma (TCGA-PRAD, Firehose Version) cohort, *LRP2* was also lower in areas of tumor, but not further lower by Gleason grade (Fig. 5C). *LRP2* did not correlate with age in either cohort (Supplementary Fig. S6A). The findings were mixed for BCR with DFKZ showing lower *LRP2* in patients who had BCR, whereas there was no difference in the TCGA-PRAD cohort (Supplementary Fig. S6B). Together, the prostate cancer data support that *LRP2* expression is highest in normal tissues and lower in tumor areas.

## Discussion

This study follows our recent finding that megalin protein is present and localized to the membrane of the prostate epithelium and that *LRP2* gene expression correlates with vitamin D status and the percentage of African ancestry (18). These findings led us to hypothesize that megalin is part of a compensatory pathway that is upregulated during vitamin D deficiency to increase the import of hormones into the prostate. Here, we focused on megalin import of SHBG-bound T, which is known to be imported by megalin. We report that megalin imports SHBG-bound T into prostate cells, is regulated by vitamin D, and is dysregulated in prostate cancer. These findings provide a direct link between vitamin D deficiency and prostate cancer disparities in AA men.

Given the pronounced role of androgens in prostate cancer risk and progression, we examined megalin as a mediator of prostate androgen levels. T is the principal circulating androgen with 70% bound to SHBG, approximately 5% free T and the remaining bound to albumin (21). T is thought to follow the free-hormone hypothesis, with only free or T loosely bound to albumin available to diffuse into tissues. We showed that megalin binds and internalizes SHBG-bound T, which is similar to other reports in LNCaP cells (21). Our work demonstrated this in two prostate cancer cell lines, in which SHBG-bound T was internalized, resulting in *KLK3* induction. We further demonstrated that the loss of megalin in mouse prostate epithelium decreased prostate androgen levels. Although knockout mice demonstrate some regulation of prostate T by megalin, it is important to note that mice differ from humans in that mice primarily circulate albumin-bound T rather than SHBG-bound T. Although megalin is a multiligand receptor that also mediates albumin uptake (36), our findings do not exclude the presence of other SHBG-T uptake receptors (37). The data shown here support megalin-dependent import of SHBG-T into the prostate and are consistent with the full *Lrp2* knockout mouse, which displays impaired descent of the testes into the scrotum, and other defects consistent with sex steroid disruption.

A negative feedback loop was previously reported for vitamin D skeletal myotube cultures, in which growth at high levels of 25D significantly decreased DBP-bound 25D uptake in cultures (38), although megalin was not specifically implicated in that report. Our findings show that 25D decreased the expression of *LRP2* transcript and megalin protein. We acknowledge that our findings present a paradox in that *LRP2* expression is regulated by the extracellular amounts of hormones, whereas we observed higher levels of androgens and vitamin D metabolites within the prostates of AA men. It was unexpected that the serum levels of hormones would correlate with *LRP2* expression rather than tissue levels. These findings reveal the complexities of endocrine hormone regulation within tissues and suggest there may be a role of membrane vitamin D receptor, which has been previously reported in other contexts (39, 40). These findings not only support a preventive role for vitamin D but also the need to avoid vitamin D deficiency in prostate cancer patients treated with antiandrogen therapies.

The presence of a compensatory feedback loop to regulate intraprostatic hormone levels by megalin has implications for the AA population who are disproportionately vitamin D deficient (41). We analyzed vitamin D metabolites in three cohorts of patient specimens. In cohort 1, which contained matched serum and prostate tissue from patients, serum 25D and prostatic DHT were significantly inversely correlated, consistent with a vitamin D-regulated mechanism for hormone import into the prostate via megalin. Moreover, AA men had higher levels of DHT in prostate tissue than EA men in cohort 1, which was corroborated in cohort 2 with additional prostate tissues. Elevated prostatic DHT may directly contribute to the increased incidence of aggressive prostate cancer and prostate cancer mortality among AA men as lower prostate androgens have been shown to decrease prostate cancer risk for incidence and mortality (42–44). AA men also presented with lower levels of serum T in cohort 1, which was validated in cohort 3 (serum only), further supporting the active transport of SHBG-T into the prostate rather than passive diffusion of T. This relationship outlines an intricate, yet detrimental interaction between the androgen and vitamin D axes that characterizes the adverse effects of this double disparity in men of West African descent.

Our finding that serum T is lower in AA men differs from previous studies that have not found racial differences in serum total T or free T (45, 46). While Hu and colleagues reported a more rapid age-related decline in serum T in AA men than in White men (47), they did not observe lower levels in AA men. Our cohorts contained one serum collection per patient, which did not show correlation between T with age. This discrepancy may reflect differences in assays, specimen preservation methods, and cohort sizes. We also showed that the relationship between serum and prostate tissue androgen levels differed between AA and EA. Although our results were significant, we acknowledge that there are limitations to our cohorts. First, there is underrepresentation of vitamin D-replete AA patients and vitamin D-deficient EA patients in the cohorts, which is needed to separate ancestry from deficiency. Second, the cohorts were made from multiple sites with imbalance between the number of patients identifying as AA or white. Although we validated the difference in prostate DHT in a second cohort of patients all from UIC, it is still rather small and larger cohorts with matched patients are needed to address these limitations.

Given the dependence of prostate cancer on androgens and the prior report of LRP2 polymorphisms with prostate cancer aggressiveness (24), we examined megalin protein and *LRP2* gene expression in patients with prostate cancer. Megalin protein levels were markedly lower in cancer tissues than in benign tissues from radical prostatectomy patients. In publicly available cohorts, *LRP2* expression was also lower in areas of cancer and further decreased by Gleason. Although these findings in the public datasets are compelling, there are several limitations including relatively small samples size and lack of clinical factors included. Because megalin expression is a feature of differentiated prostate epithelium, the levels of megalin may diminish as anaplasia increases. The consequences of reduced megalin in localized prostate cancer are yet to be determined, but it may be that those lesions are less dependent on globulin-bound hormones and utilize free hormones instead.

An important consideration regarding race-related differences is that race is a social construct and proxy for ancestry. We do not suggest that biological differences exist because of patient's self-declared race. Rather, because vitamin D status is directly correlated with skin pigmentation, our findings suggest that vitamin D supplementation may reduce the levels of prostate androgens, which would mostly affect AA men who tend to be vitamin D deficient.

In conclusion, our *in vitro*, *in vivo*, and *ex vivo* data show that megalin is functional in the prostate, transporting SHBG-bound T into the cell, which complicates the free hormone hypothesis. We also show that megalin expression is negatively regulated by vitamin D, and, in times of vitamin D deficiency, megalin is upregulated, potentially increasing the import of SHBG-T. This may signify a once-protective compensatory mechanism of vitamin D gone awry, which increases the likelihood of androgen import and increases the probability of harmful androgen actions that may contribute to the disparity in prostate cancer aggressiveness that plagues AA men.

## Supplementary Material

Supplementary Figures S1-S6 and Tables S1. Accompanies Figure 1 and 2.
S2. Accompanies Figure 2. No differences in bitransgenic mouse prostate histology, weights and fertility.
S3. Relates to Figure 3. Vitamin D receptor and androgen receptor response elements in LRP2 promoter.
S4. Relates to Figure 3. Prostate slices express hormone response components and respond to 25D and T.
S5. Relates to Figure 4. Androgen levels by Gleason and Age.
S6. Relates to Figure 6. Expression of LRP2 by age and BCR in DFKZ and TCGA cohorts.
Table S1. Cell and tissue characteristics
Table S2. Primer sequences
