# Supplementary material for: Regulation of Prostate Androgens by Megalin and 25-hydroxyvitamin D Status: Mechanism for High Prostate Androgens in African American Men
Source: Cancer Res Commun. 2023 Mar 3;3(3):371–82. doi: 10.1158/2767-9764.CRC-22-0362 (PMC9983358; doi:10.1158/2767-9764.CRC-22-0362)
Supplement: Supplementary Figures S1-S6 and Tables — S1. Accompanies Figure 1 and 2. S2. Accompanies Figure 2. No differences in bitransgenic mouse prostate histology, weights and fertility. S3. Relates to Figure 3. Vitamin D receptor and androgen receptor response elements in LRP2 promoter. S4. Relates to Figure 3. Prostate slices express hormone response components and respond to 25D and T. S5. Relates to Figure 4. Androgen levels by Gleason and Age. S6. Relates to Figure 6. Expression of LRP2 by age and BCR in DFKZ and TCGA cohorts. Table S1. Cell and tissue characteristics Table S2. Primer sequences [file crc-22-0362-s01.pdf]

Supplemental Figures

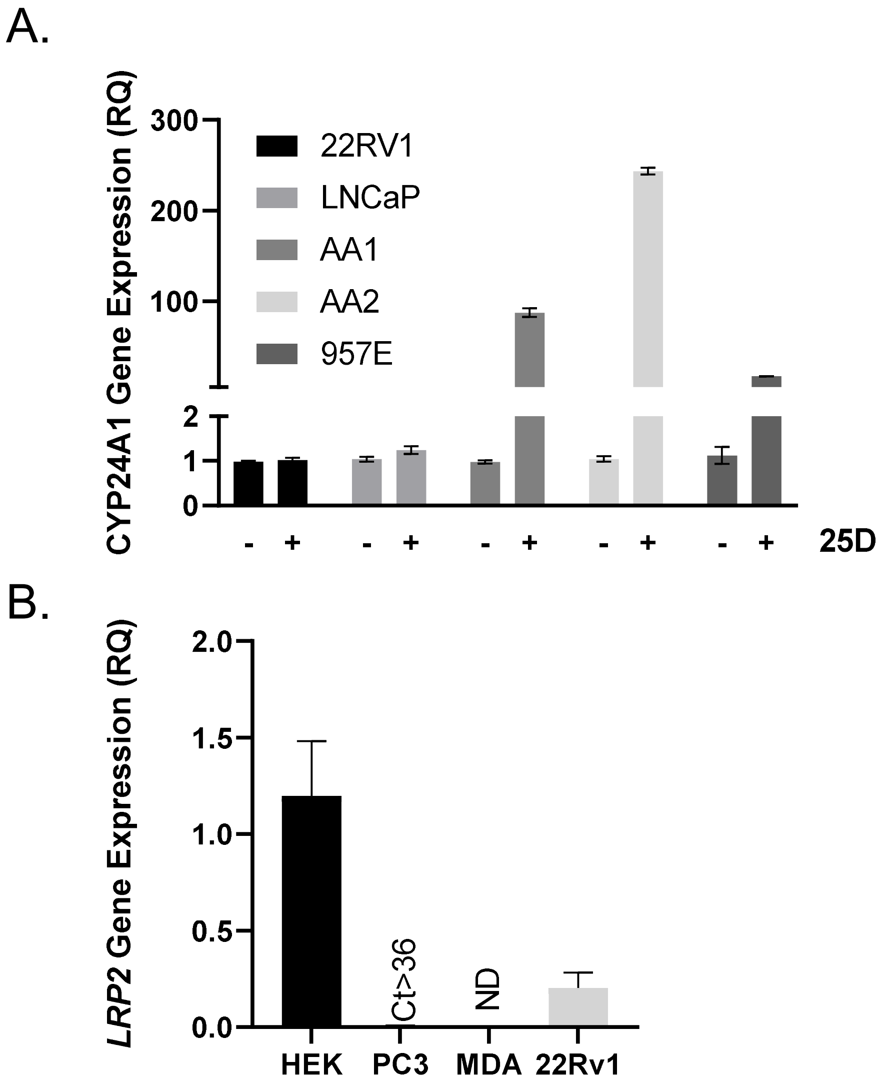

Figure S1. Accompanies Figure 1 and 2. A, PCa cells lines do not respond to 25D. RT-qPCR for *CYP24A1* following 16 hours of 50 nM/L 25D treatment in serum free conditions. **B,** PC3 and MDA-PCa-2B (MDA) cell lines do not express *LRP2*. Expression shown as mean relative quantitation to *HPRT1*. Error bars are SEM. *p<0.01

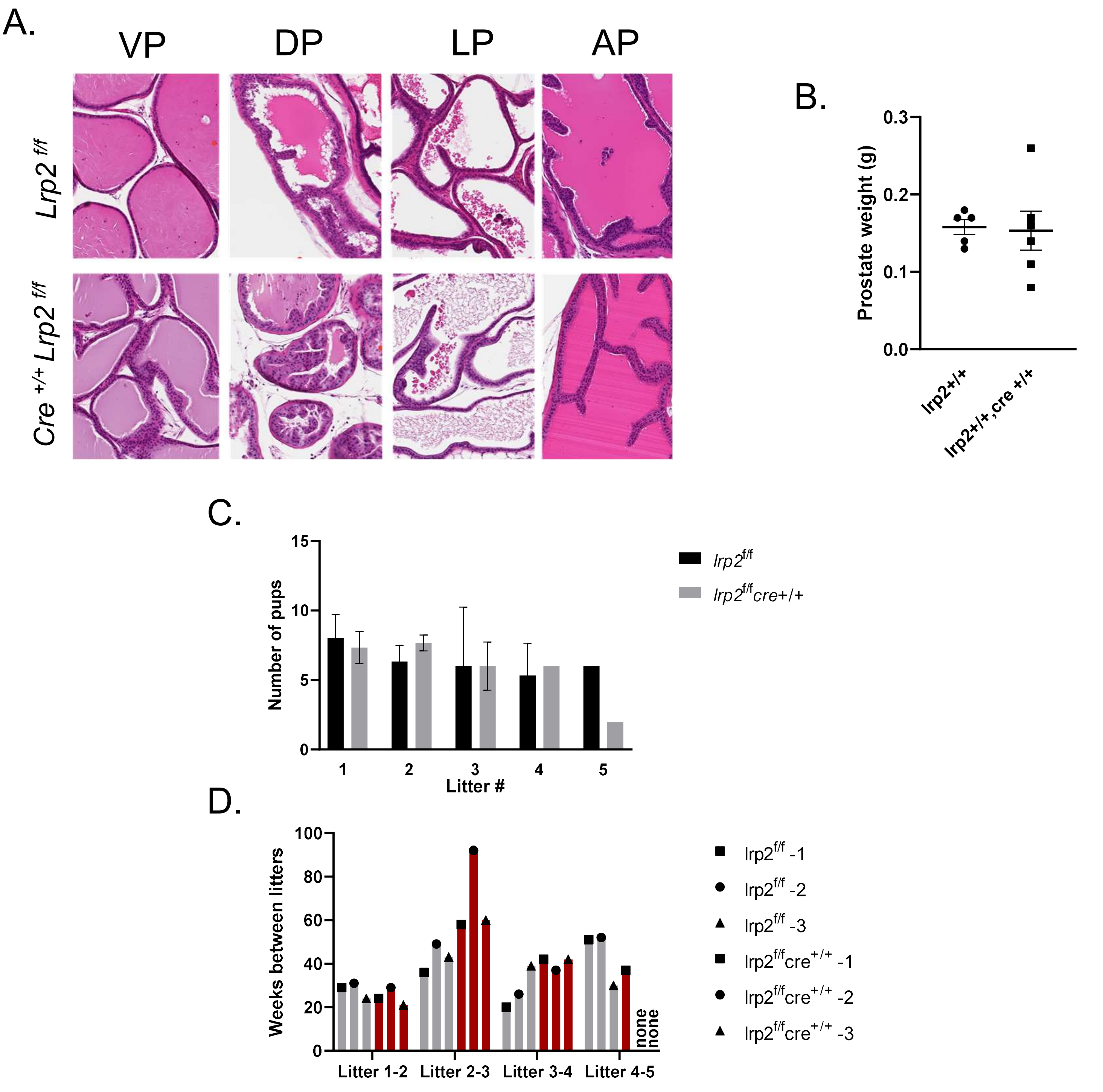

Figure S2. Accompanies Figure 2. No differences in bitransgenic mouse prostate histology, weights and fertility. A, representative H&E of prostates from 16-week old TAM-treated *lrp2$^{f/f}$* and *lrp2$^{f/f}$ cre$^{+/+}$* mice from each lobe; ventral prostate (VP), dorsal prostate (DP), lateral prostate (LP) and anterior prostate (AP). **B,** prostate weights of 3 month old male mice from *lrp2$^{f/f}$* and l*rp2$^{f/f}$ cre$^{+/+}$* mice. **C,** number of pups per litter. Note that at prior to the 5 week litter, 2 of the bitransgenic mice cages passed away from a flooded cage, so no error bars at this time point. **D,** time between litters.

A.

5'CCTGTATCCTATCAGGGGAAACCAAGATATCCGAAGGAGCCTAGAGAGATCAGGCCAGGAATGA**AGGT**

VDR1

CACAGATCTCCATCCAGCAGGCTCTTTACTCCTTCCCTTCCCTACCCCCGCCTCTTTCCCTTTCTTTCTCT

TTCCTTCATTTTTACTTGTCTGGTTGTGCTTTCCTTTTCCATTTCTTATTTTTTCTTTCTCTTCTCTATTCTTG

ACTTTCCTCCTCGTCCATTTCTTTCCATTGCTCATTTAGTAAAACTGCGTCCGTGCAGATTTCCCCGTGAG

CCGCCTCGCCGGCTTCACTTAGGAGTGCATGCGCCTGTATGAGTGCGTGTGTCTGTGCGGGGCAAACCC

TGCGGAGAGGCGAGGGCAGCGCGTGTGCACGTGTGAGTGTGCCTGTGAGAGTGTGCGCACAGGAGTGT

GCTCTTGTATGCACGCGTAAAGGGGACTGTGTATGTCGGCGTTGAAGTGTGACTGTGGAATGTGCGCGC

GTGTGTGACTG**G**CGTGTATGAGTGAATCTGTGTCAGTTGGTGTGAGAGTGTGCACACGCCTGTGTGAGT

GTCCCTGTGTGAGCACGCGTGTGTTCGAGTGCCCACTCCTCCCC**GCGCTGCAAAGTGCA**GGGGGCGGG

VDR4

CCGGGCGGCAGGGGGCCTGCCCGCTGGATTCCCGCATGCTTGTTCCG**GGGCGGTCGGGTCTA**AAGGG

VDR2

CTTTATGCACTGTCTGGAGGGTGGGGACTGGCGCGGGTAGAAAACGGGATGCCTCGGGCGTGGGGGCA

GGCTTTTGGCCACTAGGAGCTGGCGGAGGTGCAGACCTAAAGGAGCGTTCGCTAGCAGAGGCGCTGCC

GGTGCGGTGTGCTACGCGCGCCCACCTCCCGGG**GA**AGGAA**CGGCGAGGCCGGGGACCGTC**GCGGAG

VDR3

ATGGATCGCGGGCCGGCAGCAGTGGCGTGCACGCTGCTCCTGGCTCTCGTCGCCTGCCTAGCGCCGGC

CAGTGGCCAAGGTAAGAGCCCAGCCAGAG-3'

B.

Motif ID	P-Value	Matched Sequence
RXRA::VDR 1	0.000307	AGGTCACAGATCTCC
RXRA::VDR 2	0.002520	GGGCGGTCGGGTCTA
RXRA::VDR 3	0.006550	CGGTCCCCGGCCTCG
RXRA::VDR 4	0.006940	GCGCTGCAAAGTGCA

Figure S3. Relates to Figure 3. Vitamin D receptor and androgen receptor response elements in LRP2 promoter. A, -791 bp of LRP2 promoter showing mapped areas for VDR response elements. **B,** JASPAR prediction of the binding sites with P-value. Note that VDR binds VDREs as an obligate heterodimer with RXRα.

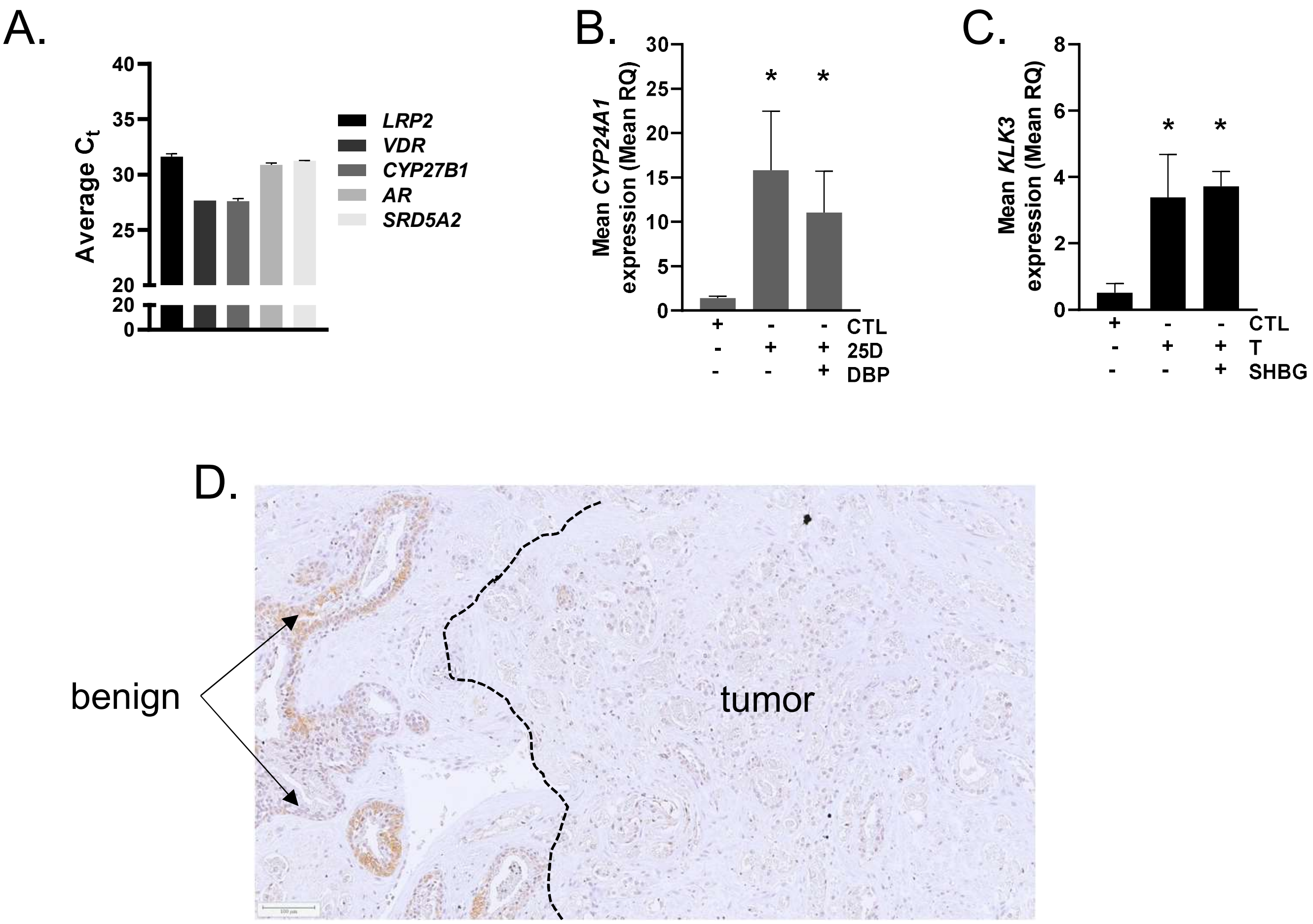

Figure S4. Relates to Figure 3. Prostate slices express hormone response components and respond to 25D and T. A, mean Ct of hormone response genes in TS1. **B**, Expression of *CYP24A1,* a vitamin D response gene, in slices treated for 24 h with 50 nM/L 25D with and without DBP. **C**, Expression of *KLK3,* an androgen response gene, in slices treated for 24 h with 50 nM/L T with and without SHBG. Data shown as mean and SEM of biological replicates from 3 patients. **D**, areas of cancer on the prostate explants had very low expression of megalin, which is consistent with Figure 5 quantitation in patient samples.

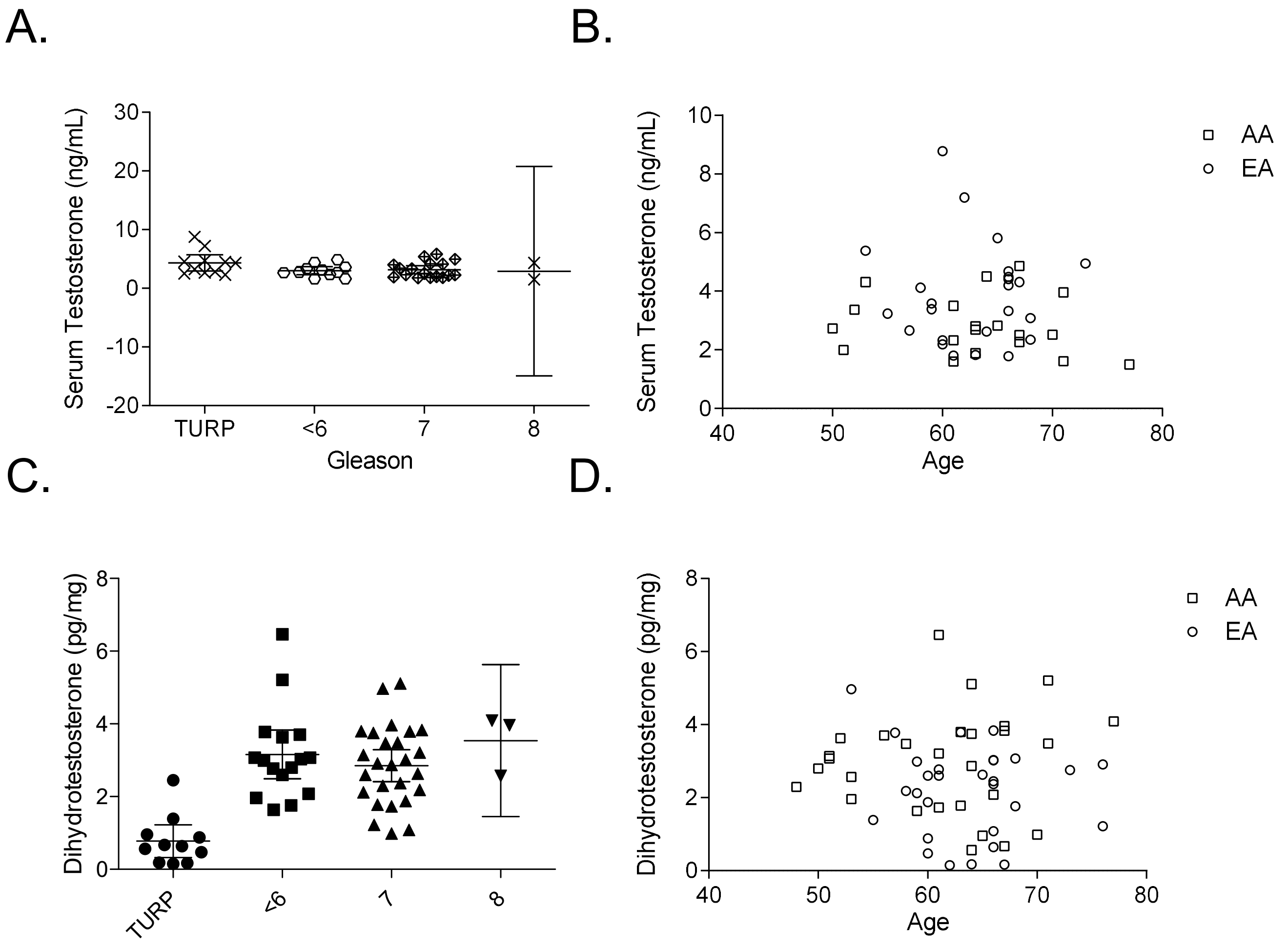

Figure S5. Relates to Figure 4. Androgen levels by Gleason and Age. A, Serum levels of T in all patients shown by Gleason sum. Benign transurethral resection of prostate (TURP) shown as control. **B**, Plot of serum T to age shows no significant correlation. **C**, Prostate DHT levels in all patients shown by Gleason sum. DHT was measured in benign areas of prostates and the Gleason score is from the tumor within the same prostate. TURP shown as control. **D**, Plot of prostate DHT to age shows no significant correlation.

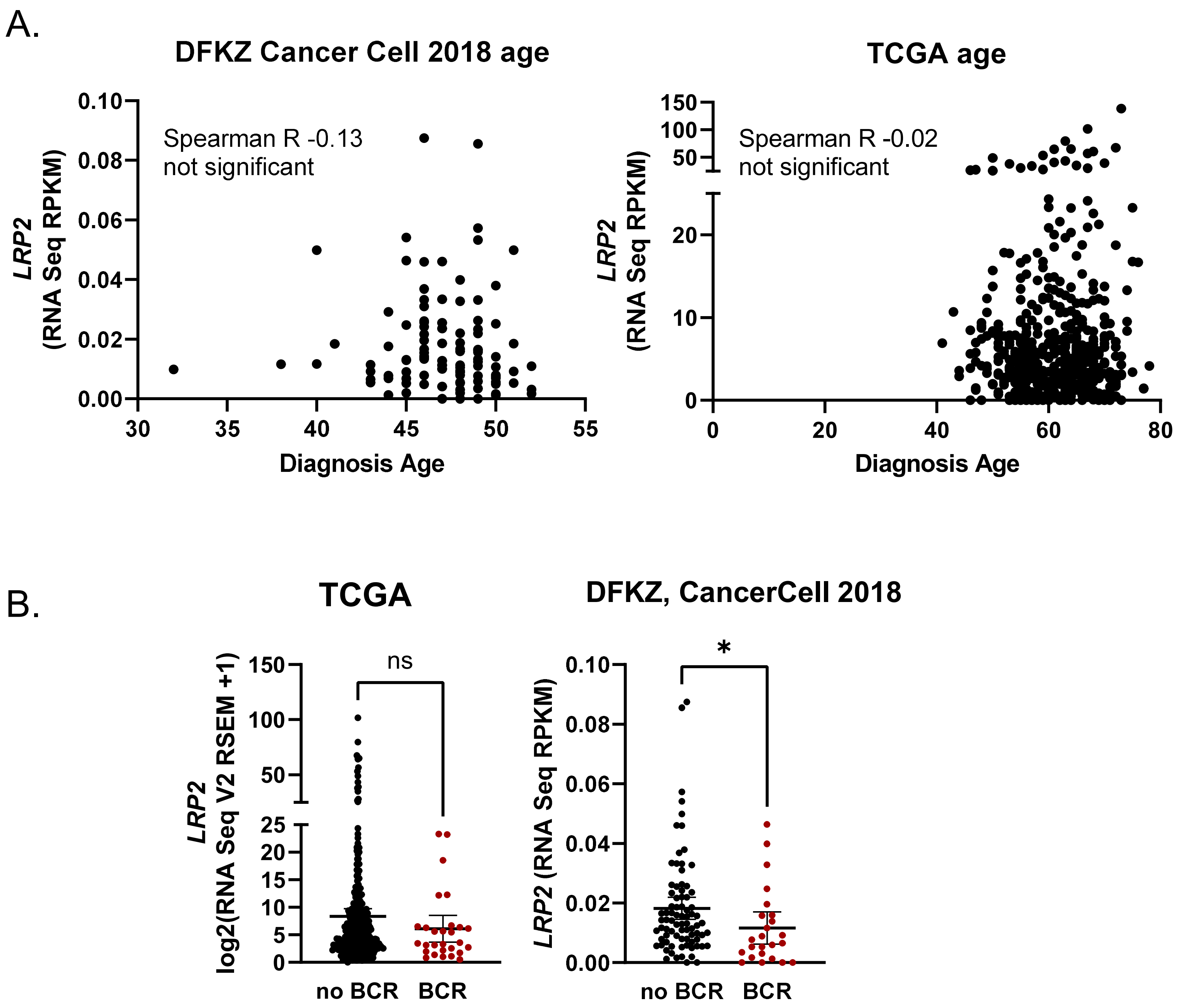

Figure S6. Relates to Figure 6. Expression of *LRP2* by age and BCR in DFKZ and TCGA cohorts. A, *LRP2* expression does not correlate with age. **B**, mixed results for *LRP2* expression and BCR in TCGA and DFKZ cohorts. Analyses on Cbioportal.

Table S1. Cell and tissue characteristics

MODEL	TISSUE	MORPHOLOGY	DISEASE	ETHINICITY
**PrE-AA1**	Prostate	Epithelial	Benign	African American
**PrE-AA2**	Prostate	Epithelial	Benign	African American
**957E-hTERT**	Prostate	Epithelial	Cancer (local)	White European descent
**HEK293**	Kidney	Epithelial	Benign	Unknown
**22Rv1**	Prostate	Epithelial	Malignant origin Behaves benign	White European descent
**LNCaP**	Prostate	Epithelial	Cancer (lymph node)	White European descent
**PC3**	Prostate	Epithelial	Cancer	White European descent
**MDA-Pca-2B**	Prostate	Epithelial	Cancer	African American
**TS-AA1**	Prostate	Epithelial	Benign	African American
**TS-H1**	Prostate	Epithelial	Benign	Hispanic

Table S2. Primer sequences

SPECIES	GENE	FORWARD (5’ -> 3’)	REVERSE (5’ -> 3’)
HUMAN	***LRP2***	CTCCTGGGTGTGTGACCAA	CATCGGGGCAGTCTCTGAC
	KLK3	TCCAATGACGTGTGTGCGCA	CCTTGATCCACTTCCGGTAA
	CYP24A1	GGCAACAGTTCTGGGTGAAT	ATTTGAGGACAATCCAACA
	CYP27B1	TTTGCATCTCTTCCCTTTGG	CTCAGGCTGCACCTCAAAAT
	VDR	GACCTGTGGCAACCAAGACT	GAACTTGATGAGGGGCTCAA
	SRD5A2	ATATATTGCGCCAGCTCAGG	GGAAATTGGCTCCAGAAACA
	AR	TTGTGTCAAAAGCGAAATGG	CAATGGGCAAAACATGGTC
	RPL13A	GGATAAGAAACCCTGCGACA	CTCGACCATCAAGCACCAG
	HPRT1	TGCTGACCTGCTGGATTACA	CTGCATTGTTTTGCCAGTGT
MOUSE	***Pb-MerCreMer*-mutant (genotyping)**	ACGACCAAGTGACAGCAATG	TAAGCAATCCCCAGAAATGC
	***Pb-MerCreMer*-wild type (genotyping)**	CTAGGCCACAGAATTGAAAGATCT	GTAGGTGGAAATTCTAGCATCATC
	Lrp2-Flox* (genotyping, *wt = 206bp, flox=320bp)	CACCATGTCCCTCAGTTGTG	CACCATGTCCCTCAGTTGTG
	***Lrp2 exons 71-75* (recombination)**	CACCGTTGAGTGTGATGCAG	CACCGATGTCCATGTTCACA
	***Lrp2 exons 76-77* (control)**	CTGGTTTTCCACATTTTCTGG	CTGGTTTTCCACATTTTCTGG
